# Gender Inequality in Precarious Academic Work: Female Adjunct Professors in Italy

**DOI:** 10.3389/fsoc.2019.00087

**Published:** 2020-01-17

**Authors:** Gianluca De Angelis, Barbara Grüning

**Affiliations:** ^1^Department of Sociologia e Diritto dell'Economia, University of Bologna, Bologna, Italy; ^2^Department of Sociologia e Ricerca Sociale, University of Milan Bicocca, Milan, Italy

**Keywords:** unpaid work, adjunct professor, academic career, gender inequalities, Italy

## Abstract

International research studies and national reports point out two specific aspects which characterize women's academic careers (cf. Eagly, 2003; Glass and Cook, 2016). First, few women advance to senior academic roles. Second, although female academics progress in numbers equivalent to their male colleagues up to a certain point, in most cases their academic career paths either stop before they arrive at tenured positions or they remain in the lower ranks of the hierarchical academic structure. Thus, while the numeric growth and temporal extension of fixed-term positions has, overall, increased women's opportunities for researching and teaching at universities, on the other hand, it has impeded their access to tenured positions. To better highlight this dynamic, this article focuses on the situation of female adjunct professors in Italy. The interest in adjunct professors is twofold: on the one hand, the social and economic status of adjunct professors in the Italian academic system have worsened over time, from independent to formal independent workers; on the other hand, compared with other non-tenured positions, there are substantially fewer female adjunct professors than male. We first provide an overall picture of the historical and juridical transformations of the rank distribution of faculty in Italian universities from the perspective of gender. As a second step, we compare the actual working conditions of female and male adjunct professors on the basis of a survey carried out from January to October 2018 (5,556 respondents corresponding to more than 20% of the population) and semi-structured interviews with 31 adjunct professors. The aim of the analysis is to pinpoint objective and subjective gender similarities and differences regarding both socio-economic variables and the ways male and female adjunct professors think about their academic and extra-academic work; how they experience the academic environment between paid and unpaid work, construct their professional/academic identity, and imagine their professional future and perceive problems related to the administration and organization of their academic work.

## Introduction

The focus of this article are the working conditions and academic aspirations of female and male adjunct professors (APs), as paradigmatic example of a new form of precarious working conditions in the academia^1^. Thus, in light of the existing studies on the relationship between non-standard work arrangements and social inequalities (cf. Standing, 1989, 2014; Bigi et al., 2015; Krinsky and Simonet, 2019), our main interest is to explore how social gender inequalities are produced and practiced in the Italian academic field (cf. Bourdieu, 1984; Murgia and Poggio, 2018), on the basis of its genesis and social structure. More specifically, by choosing adjunct professors as research object we intend to point out two specific questions concerning the transformation of the Italian academic structure in the last four decades. The first one regards the increasing use of adjunct professors as disguised form of self-employment in the academic system, in front of a progressive public disinvestment in the Italian Universities, especially for teaching activities. The second one regards the low prestige (symbolic capital in Bourdieusian term) of adjunct professors also with respect to other precarious academic positions, such as the research fellows and assistants. Thus, most of the adjunct professors are men. This statistic evidence puts in question the various researches on gender inequalities in Academia, which highlight how the so-called “academic housework” is mainly carried out by women (cf. Heijstra et al., 2017). For examining how gender inequalities are produced in academia, our case study shows us that beyond taking into account the different degree of prestige of the various academic activities, further categories are to consider, inherently to the specific historical and social context under investigation.

Then, to investigate our topic, three theoretical premises are needed.

The first regards the ongoing diffusion of non-standard work arrangements in the intellectual field, i.e., the market of symbolic goods (Bourdieu, 1993). The intellectual field and its specific subfields (for example, the literary, artistic, and academic subfields)^2^ have always been places of economically unstable working conditions. Nevertheless, the progressive institutionalization of these subfields has not only radically transformed the social conditions which reproduce precarious positions, but has also been supported by new discourses and rethorics legitimizing the existence of these conditions. The *second* premise regards the organizational and cultural changes which have affected higher education in the last three decades as a result of the progressive development of new forms of “academic capitalism” (Slaughter and Rhoades, 2004). For our context, one of the main consequences is the increasing centrality of research activities (and output) for evaluating the efficiency of academic actors (institutes and academics) and increasing academic reputations (cf. Deem and Lucas, 2007; Arimoto, 2015), which has come at the expense, however, of the importance given to teaching activities (Fuller, 2009, p. 25; cf. Lyotard, 1979). Finally, the third premise regards the different impact of precarious working conditions on the career paths of women and men in intellectual professions, by worsening existing disparities in the gender structure of academia (cf. Hirsch and Leppel, 1982; Menges and Exum, 1983; Pool et al., 1997; Blickenstaff, 2005; Fox, 2005; Bataille et al., 2017; Heijstra et al., 2017; Argenvall and Beach, 2018; cf. Murgia and Poggio, 2018).

In light of this framework, our hypothesis is that female APs have more difficulty than male APs in pursuing an academic career. This hypothesis would also explain, why female APs are fewer and younger than male APs, that is they are more disposed to abandon their academic path. For supporting this hypothesis we relate the concept of career as developed by Hughes (1958) with the concepts of (academic) field, habitus, and capitals as developed by Bourdieu (1979, 1984, 1986).

For what concerns Hughes' concept of career we first considers the distinction he stresses between an objective understanding of career as ≪a series of states and clearly defined offices≫ and a subjective understanding of career as ≪the moving perspective in which the person sees his life as a whole and interprets the meaning of this various attributes, actions, and the things which happen to him≫ (Hughes, 1958, p. 63). With respect to our study the question is twofold. On the one hand, it regards the objective effects which the “de-standardization” of the academic career (Bataille et al., 2017) has on the academic working conditions and structure, as the increasing use of different forms of fixed-terms and non-standard contracts. On the other hand, it regards the different ways of experiencing and interpreting the “de-standardization” processes of the academic career by different social groups, i.e., in our case the female and men APs. As Hughes underlines, the career is ≪by no means exhausted in a series of business and professional achievements. There are other points at which one's life touches the social order, other lines of social accomplishment—influence, responsibility, and recognition≫ (Hughes, 1958, p. 64). The latter statement highlights how in the academia there are different (socio-spatial) degrees of “social order” referring either to the workplace(s) or to the wider local academic community or to the (national and international) disciplinary community. Thus, the position an adjunct professor occupies within these social orders depends not only on his/her official tasks and duties, but also on the content and form of his/her social relationships in the different professional settings and networks. In other words, following Bourdieu, we may argue that the position APs occupy within the academic field (in its different socio-spatial dimensions aforementioned) depends not only on their contracts (i.e., as indicative of their economic capital) and academic qualifications (scholastic cultural capital, cf. Bourdieu, 1986), but also on their social capital.

The social capital one possesses—which is indicator of the integration in both the everyday life activities of the specific institute and in the broader (inter)disciplinary scientific community—influences in turn the ways of perceiving own position and image own trajectory in the field itself. Not least it influences the building of a specific academic habitus, which vice versa, influences the ways of acting, interacting and building social relationships. Furthermore, in the case of the APs it is to consider that their economic and symbolic capitals depend also on the gain and prestige of their extra-academic activities. The sum and entanglement of these factors conditions the time perception and time budget of the APs. As Heijstra et al. (2017) have already noticed, time is a crucial resource for cumulating academic capital and also one of the reason of the gap between female and male academics. We can then consider time as a resource in two ways. First, time is needed for carrying out and conciliating different professional and academic activities in the everyday life. Second, time is needed for transforming one form of capital (cf. Bourdieu, 1986), in particular the economic one, in other forms of capital, i.e., the social and the scientific ones. In other words, the economic safety provides the condition for thinking long-term strategies, for cultivating social relationships and publishing. This entails, not least, to consider how the different “contingencies of a career” (Hughes, 1958, p. 130) influences also the capability of projecting oneself in the future, predicting about the course of the events, and taking crucial decision (Hughes, 1958, p. 28–29). In Bourdieusian terms, the contingencies of a career influence the way the academic habitus, the ≪structuring structure, which organizes practices and the perception of practices≫ (Bourdieu, 1979, 1984, p. 170) is built by female and male APs.

Summing up, through the analysis of the empirical data, we will argue that in Academia gender inequalities are a field effect, depending on the different economic, cultural and social resources male and female academics possess for constructing their own strategies both in their everyday life and for the future.

In what follows, we first discuss how, nowadays, discourses around the importance of “subjectivity” in the new labor market represent a new source for legitimating non-standard work arrangements, especially in those sectors which are either traditionally closer to women's working activities (cf., Fürth, 1906; Weber, 1913) or related to intellectual activities. As a second step, we sketch the main transformations of the Italian academic structure over the last four decades, in light of two international trends: (1) the increasing separation of teaching and research in the organization of academic life, with consequences on the structuring of the academic paths of individuals aspiring to academic careers; (2) the increasing weight of a “technical control” over the academic work (Miller, 1995) within universities that follows the logic of the private market.

Within this frame, we will try to better highlight the different academic career paths of women and men in Italy from a longitudinal perspective. In this regard, the fourth and fifth sections are devoted to the analysis of our empirical data on APs in Italy. Thus, on the basis of a survey carried out from January to October 2018 (5,556 respondents corresponding to more than 20% of the population) and semi-structured interviews with 31 APs, we will compare the actual working conditions and aspirations of female and male APs. The aim of the analysis is to pinpoint objective and subjective gender similarities and differences regarding both socio-economic variables and the ways male and female APs think about their academic and extra-academic work; how they experience the academic environment, moving between paid and unpaid work, construct their professional/academic identity, imagine their professional future, and perceive problems related to the administration and organization of their academic work.

## Subjectivities at Stake: The Other Side of Non-standard Work

The diffusion of non-standard work arrangements provides a key perspective for understanding the ongoing transformations in the labor market; however, attempting to catch these transformations through statistical observations risks losing communicative effectiveness because people tend to have difficulty recognizing the peculiarities of their own working conditions in terms of the categories defined by the researchers, which necessarily objectify not only the working conditions but also the subjective dimension of the working experience. Taking this question into account can be crucial not only in investigations for scientific purposes but also in inquiries moved by a pragmatic worldview which aim at influencing the orientation of either specific policies (i.e., active employment policies) or trade union campaigns.

Various scholars have observed how, when analyzing statistical indicators, researchers tend more to construct than describe the reality under investigation (Desrosières, 2010, 2011). In particular, Robert Salais claims that when researchers use a table to represent data, they adopt specific *conventions of equivalence* which determine what can be considered as similar (Salais, 2009, p. 118). When we consider, for example, the employment rate, we look at all the people who have a job in a certain timespan. But to what extent can we assume that people in similar positions in the labor market feel that they share the same conditions? With respect to the distinction between “employed” or “unemployed,” these people have a different status, but we do not know anything about an employed person's job, whether, for instance, it is a part-time or low-wage job which could make his or her life more similar to the lives of the unemployed. In the current labor market, these questions have become increasingly important since they highlight how the idea of human subjectivity is at the core of both non-standard work arrangements and new working methods.

On the other hand, it would be erroneous to believe that the centrality of human subjectivity in the contemporary labor market is a prerogative of post-Fordism or an effect of the diffusion of non-standard work arrangements. As Gramsci had already argued in the 1930's, one of the pivotal aspects and innovations of the Fordist production system was the “creation” of a new human being (Gramsci, 1978). Indeed, Fordist organization needed reliable workers who were able to work without interruption. In this sense, new working methods were introduced which, according to Gramsci, followed “puritan policies” which also applied outside the workplace:

“Puritanical initiatives simply have the purpose of preserving, outside of work, a certain psycho-physical equilibrium which prevents the physio-logical collapse of the worker, exhausted by the new method of production. This equilibrium can only be something purely external and mechanical, but it can become internalized if it is proposed by the worker himself, and not imposed from the outside, if it is proposed by a new form of society, with appropriate and original methods” (Gramsci 1939).

In this regard, Fordism can be seen as a game whose rules are embodied by players to the extent that they forget that it is a game. This equilibrium is very close to Bourdieu's idea of *illusio*, defined as ≪the enchanted relation to a game that is the product of a relation of ontological complicity between mental structures and the objective structures of social space≫ (Bourdieu, 1998, p. 77).

From this point of view, the project of Fordism was ambitious. For a new society based on a specific production regime, a simultaneous effort should involve both the productive sphere, thanks to which the workers earned their wages, and the re-productive (or non-directly-productive) sphere of life, in which the workers and their families spent their wages. According to the dominant discourse, the conditions of most workers were justified by the fact the workers could fulfill themselves outside the production sphere. Clearly, this project was based on an intrinsic gender discrimination. While the men of the Industrial Revolution were the “breadwinners” for their families, women's wages, if present, could not be higher or more significant than a complementary resource (Zelizer, 1997; Bellavitis, 2018) and, ≪At the same time, women, identified as “nature,” were excluded from the “public” space of politics, reserved for men≫ (Bellavitis, 2018, p. 10).

In this regard, we can argue, the bourgeois ideology of the family and the separation between the private and public spheres (cf. Weber, 1921; Sennett, 1977) had deeper consequences for women than for men. While for men economic wages were an objective measure of recognition, for women recognition was mostly symbolic and related to their subjective abilities and skills. As the first female sociologists active between the ninetieth and twentieth centuries observed, care activities outside the domestic sphere were rarely considered an objectified form of work by men and, in this regard, such jobs did not deserve the same recognition as the “traditional” objectified male jobs (cf. Weber, 1913). Hence, the realization of the “natural” relational activities of women as mothers and wives outside the private sphere made visible the condition of women's work (and “female” work), but without this work being recognized as “real” since it was not directly “productive” (cf. Simmel, 1902; Delphy, 2004; Simonet, 2018).

In a similar way to the case of women's work in the earlier Fordist era, nowadays many jobs which manifest a subjective dimension are considered “non-productive” activities. Discourses around the vocation and passion of workers in particular mask the objective structures of many employment markets in order to legitimize non-standard work arrangements in which, for example, people perform the same tasks but under unequal contracts and working conditions, or are engaged in gig-jobs or unpaid work. From a constructivist perspective (cf. Berger and Luckmann, 1966; cf. Knoblauch, 2009), we can point out three processes: the internalization, externalization, and objectification of the idea of “vocation.” Whereas, the internalization of the idea of vocation is accomplished by the processes of typification and socialization, which mainly concern the everyday dimension, the processes of externalization and objectification of the idea of “vocation” result in the institutionalization and legitimization of non-standard work arrangements.

Thus, the internalization of “vocation” depends on the ways people re-signify and legitimize their work in terms of its originality and innovative qualities (cf. Heinich, 2008). We can notice an upside-down rationality at work here (Bourdieu, 1998) whereby, following Heinich's observation about writers, such people do not work to earn a living, but rather earn a living in order to carry on certain activities (Heinich, 2008, p. 1) which, while providing distinctiveness, also justify precarious working conditions (cf. Giancola et al., 2016). In this regards, as Richard Sennett suggests, vocation can be seen as a *sustaining narrative* (Sennett, 2008, p. 263–65), that is, a narrative which supports one's own professional identity from the outside and presents a typified structure and form. On the other hand, the objectification of “vocation” goes through socialization processes mediated by key socialization agents or intermediaries. In *Contribution à une sociologie de la vocation*, Suaud observed, for instance, how the clergy played a pivotal role for the inception of the concept of a religious vocation. Thus, according to Suaud, religious dispositions, which are usually perceived as something exclusively individual, actually depended on how clergymen contributed to forming the perception and thinking schemes through which laymen developed a religious habitus, until a religious career was considered the most desirable of careers (Suaud, 1974).

As in religious and artistic contexts, in many work sectors the precariousness of working and living conditions strengthen the feeling of predestination and vocation. What matters, however, is not the subjective tension itself, but the forces which create this tension. Whereas, for artists and craftsmen the work activity justifies the working conditions, in other careers, such as religious ones, working conditions are justified by something (or someone) that goes further than the “objective reality” of the work itself. Thus, if on the one hand we may agree with Sennett when he highlights how, in the new spirit of capitalism, there is no place for the strong passion the craftsman has for his work (Sennett, 2006, 2008), on the other hand, we can observe how, in certain productive fields, work is represented as an opportunity to realize something greater than the work itself. This is the case in several sectors, including social work (De Angelis, 2017), intellectual work, and work in the broader cultural field (Armano and Murgia, 2012, 2013). In these sectors, the expression of passion and subjective meanings are considered a means of good production, and this “whip of the beyond” (Rastello, 2014) has therefore become a breeding ground for the roots of neoliberal subjectivity (cf. Illouz, 2007).

In the following section, before exploring the deeper insights of our research findings to see how this tension between the subjectification and objectification of working conditions differently structure the academic work of female and male APs, we will try to shed light on how the international trends of “academic capitalism” have taken root in Italy.

## Academic Careers in the Neoliberal University

In 2008, while investigating how younger academics constructed their professional identities, Louise Archer pinpointed the emergence of a neoliberal subject (Archer, 2008). To better illustrate the difficulties younger academics encountered in trying to keep up with the increasing expectations of the new Public Management logic which rules the academic system, she discussed an excerpt from her interview with Rose^3^. In the interview, Rose stated she began working early in the morning because she had difficulty sleeping due to anxiety. She knew that other colleagues preferred working in the morning, but she tried not to give weight to the fact that she did it as well because she considered it a free choice. Thus, she felt that it was her choice and, in a certain sense it was, but to what extent was she really free to choose?

Actually, Rose's words give a precise description of how neoliberal rules have been internalized. With Foucault, we can say that the power of the neoliberal *apparatus*^4^ consists in shaping the choices of individuals (Foucault and Gordon, 1980; Foucault et al., 2004; see also: Agamben, 2006; Dardot and Laval, 2013) even to the extent, however, of affecting their psychologic health (Ehrenberg, 1999; de Gaulejac, 2009). To come back, instead, to the Bourdieusian framework (cf. Bourdieu, 1984; Bourdieu and Wacquant, 1992), we can observe how the new neoliberal logic of the academic field has shaped a new academic habitus by redefining the everyday working practices of younger academics, so that the new rules of the game and evaluation system appear “natural” to them and, in this sense, more difficult to criticize. Thus, differently from her older colleagues, Rose and her younger colleagues work in a more and more market-oriented context. Nevertheless, while the evaluation dynamics press them to maintain high productivity standards, maintaining those standards guarantees neither career success nor the timing of this eventual career. In other words, meeting these standards enables access to the competition and, implicitly, the legitimation of one's results, but does not assure the achievement of specific career outcomes.

The case studies carried out by Archer on younger academics of UK universities is paradigmatic of how the diffusion of neoliberal culture in the last 30 years has had a more global impact on the academic system, at least that of Western countries. In particular, the example of Rose well illustrates how structural changes condition the space of possibilities for constructing career paths and, in turn, affect the strategies, practices, and aspirations of individual actors in the academic field, even modifying their ways of perceiving and thinking about their academic activities and identities.

Turning to the Italian situation, we can observe how neoliberal culture has also taken root here, though it has been adapted to the specific logic and structure of the Italian academic field. One aspect which is important to mention here, and which may also help us in the interpretation of the empirical data, is the tension existing between the central organization of the academic system under the control of the State and local centripetal forces acting as “academic tribes” or “clans” (cf. Deem and Lucas, 2007; Capano and Meloni, 2009) which often prevent the implementation of both national reforms and international standards.

Let us start by sketching the principal changes regarding the academic ladder and careers in Italy since the university reforms of 1980 in order to gradually introduce the question of how academic teaching and the role and tasks of precarious academic staff involved in teaching activities have changed in the last four decades (cf. Moscati, 2001).

Before 1980, the academic structure revolved around a full professor, who was also the holder of a chair. Under him/her, there were several positions devoted mostly to teaching, positions which have been progressively reformed or suppressed over time^5^. Typically, after receiving a degree, a young scholar could accede to the “volunteer assistant” position. After obtaining the so-called *libera docenza* (a qualification permitting one to teach at university), he/she could become an *assistente ordinario* (who provided research and teaching support to a full professor) and then, when a post was available, *professore incaricato* (who was responsible for teaching a specific course, along with all related exams and theses, for one academic year). After several years, usually spent in different universities^6^, the *professore incaricato* could become a full professor and return to his/her original university. In order to understand the composition of university staff before 1980, the annual statistical report of the education department in 1970 identified, in addition to 2,347 full professors, 2,394 *professori incaricati*, 6,556 *liberi docent*, and 15,987 “volunteers” (ISTAT, 1971).

The university reform of 1980 radically restructured the existing formal academic ladder into three levels (full professor, associate professor, and lecturer) without, however, modifying the recruitment process which strictly depended on informal dynamics, generally based on familial power relationships. This reform also introduced the figure of the *docente a contratto*^7^ (adjunct professor, AP). Similarly to the *libero docente*, APs stipulated a private contract with the university. But, differently from the *libero docente*, APs were selected for the professional expertise they had developed outside of academic institutions. As a result, their classes were understood by law as electives supplementing the courses of the standard curriculum.

The State's strong investment in economically supporting the reform made possible the hiring of about 30,000 people as lecturers and associate professors in just 5 years. As a result, it seemed to the legislators that the role of APs could remain “marginal” to the functioning of university teaching activities, while also assuming that, for APs, collaborating with the university would be a sign of prestige.

In the 1990s, growth in the recruitment of new academic staff did not keep pace with the increasing number of students. From 1983 to 1998, the student/professor ratio increased from 24.2 to 29.0. In order to find a solution to the lack of teaching staff, in 1990 Law no. 341 (art. 11) established the possibility for lecturers to teach courses (for no more than 60 h per academic year). Nevertheless, this measure was insufficient given the ongoing changes in the organization of the courses carried out over subsequent years, in particular the increasing autonomy of academic institutes in establishing their own degree courses and the inception of two levels of undergraduate and specialization courses (Law no. 509/1999). Thus, in 1998, the Minister for Universities, through a further decree, Law no. 242/1998, established that APs were now permitted to teach required courses. Despite the increasing teaching needs, it does not seem that the university policies were aimed at giving a structural answer to the problem. Since 1998, the teaching shortage has been mostly covered by counting on the willingness of lecturers (who until 2010 were not paid for lecturing), increasing the teaching duties and responsibilities of both APs and associate and full professors, and increasing the number of APs.

On the other hand, since the 1990s, the teaching and research activities of professors have become increasingly subject to evaluation (cf. Moscati, 2001; Rebora and Turri, 2011). Nevertheless, whereas evaluation systems concerning teaching activities are mainly oriented toward checking that teachers have fulfilled their duties, the evaluation systems for research activities are oriented toward rewarding those who have “the best impact factor.” What matters for our concern is not so much that in both cases the research and teaching capabilities of academics are objectified, according to specific standards that appear to be “neutral.” What matters is, rather, that this dual system of evaluation has created an implicit hierarchy between teaching and research activities, so that the former are more and more considered only as a duty, whereas the latter are a source of prestige. The gap between research and teaching duties has increased since 2010 with the new reform of Law no. 240/2010 which introduced the “*abilitazione*,” a national qualification needed to become an associate or full professor, that is essentially based on the evaluation of scientific output (i.e., scientific articles, monographic works, book chapters, patents), excluding teaching experience as relevant criteria.

Summing up, the reform processes carried out during the last 40 years have had two main consequences. The first is the increasing divergence in terms of the rights, social recognition and career opportunities between those who are employed with fixed-term research contracts and those who are mainly employed with fixed-term teaching contracts^8^. The second consequence concerns the increasing split between those employed with fixed-term contracts in the so-called “hard” (scientific) and “soft” (humanities) disciplines, not least because the former have greater possibilities of finding institutional and external research funding. In this second case, the question is not so much whether they may have different career prospects, but rather that they cultivate a different idea of “the University,” its mission and social and cultural tasks. This rift has clearly surfaced during the meetings organized by the FLC-CGIL trade union and other independent academic associations on the occasion of their campaign against academic precariousness in Italy (May 2018–October 2020). Thus, those who belong to “hard” disciplines consider teaching mainly as a non-prestigious, time-consuming activity that is unhelpful for their careers. Conversely, those who belong to social science and humanities (SSH) disciplines are more concerned with problems regarding the organization of teaching and the teaching duties of academic staff. This does not mean that SSH-scholars are exonerated from the competition based on publishing and research duties. It means, rather, that, in the SSH-disciplines, teaching is often one of the few possibilities scholars have for continuing to work at the university. As a result, such scholars need to work more than others.

In addition to this cultural variable, the impact of two further socio-structural variables on the working conditions and social status of APs need to be considered: age and gender. Younger APs usually have more available time than older ones to spend on teaching activities, but also less economic stability; in addition, male APs may count on a more comfortable labor market and are less embedded in family duties than female APs. Thus, age and especially gender create conditions of inequality, even if these conditions of inequality exist prior to the organization of academic work. Nevertheless, in light of the specific social structure of the academic field, these inequalities take on specific significance, which especially emerges when we look at the temporal dimension of the academic life of precarious academics, partially highlighting how they are generated at the early stage of an academic career, when they are almost invisible (see on the topic: Murgia and Poggio, 2018).

If we look at the percentage of women at the different stages of the academic career ladder (from “research fellows^9^” to full professors) from a longitudinal perspective, we notice that, in the last two decades, whereas the number of female and male research fellows is almost equal (in some years, the number of female research fellows was higher than the number of male research fellows), the number of women in tenured and tenure-track positions over time decreases, decreasing at a higher rate the more prestigious the academic position is. This phenomenon also concerns the new tenure-track position of junior professor introduced in 2010. Two explanations are here possible. The first one is that recruitment is higher in male-dominated disciplines. Nevertheless, data on junior professors collected in April 2019 highlights how, with the exception of life sciences and the arts, all other disciplinary areas are predominantly male (data source: CINECA). The second one is that the number of female research fellows is higher in the early phase of academic careers and decreases progressively in the following years (González Ramos et al., 2015; Komlenac et al., 2019). As a result, according to this interpretation, the number of women who can compete for tenure-track positions is inferior to the number of possible male competitors. The trends of male and female APs in the last two decades are conversely very different from those concerning research fellows. Indeed, over time, the number of female APs has been consistently about 30 percentage points lower than the number of corresponding male colleagues (source: USTAT-MIUR).

In the next section, we will see how this difference in gender distribution among research fellows and APs over time probably depends on two factors: the fact that the average age of APs is higher than the average age of research fellows, and that most APs work more than one job. It seems, then, that for women, working and living as APs is possible up to a certain age. A key question we will try to tackle more widely through the data analysis regards to what extent the structuring of precarious conditions in the academic career path mostly prevents women's access to academic careers.

## The Careers of APs: A General Overview

**Figure 1:**
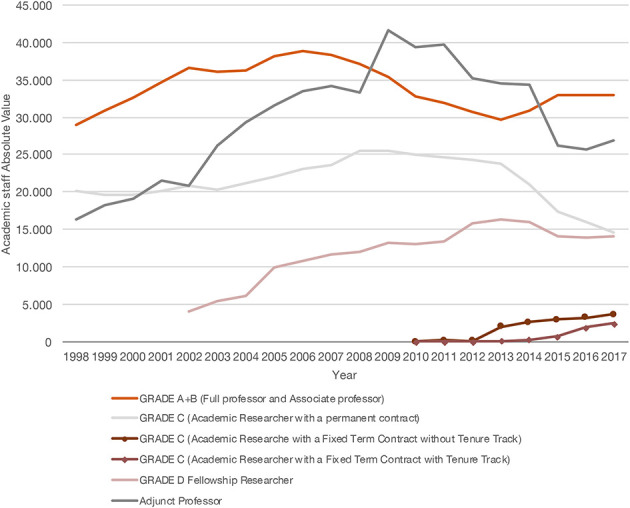
Academic staff contract, absolute value by year (Source: MIUR).

As mentioned above, the figure of the AP was established in 1980. Nevertheless, its growth became numerically significant only after 2003, we can claim, for three main reasons. First, the application of the degree course reform which started in 2001 (corresponding to the Bologna process) replaced the old “*laurea*” (4 or 5 years) with two levels of (undergraduate) and (specialization) degree courses. Second, the new law regarding university autonomy (Law no. 509/1999) gave universities the ability to more independently establish new degree courses and *curricula*. Third, the new wave of recruitment after 2001 concerned tenured researchers (RTI) without teaching duties, whereas the number of full and associate professors after 2003 decreased consistently. In 2009, with the block on replacing positions lost to turn-over, the number of APs peaked at 40,000. From 2009 to 2013, the number of APs remained higher than the number of full and associate professors together. In recent years, the decrease of AP positions has mostly depended on a special plan (2013–2014) for the professional advancement of the old RTIs to positions as associate professors. Conversely, the number of junior and senior researchers, the two new figures which were introduced in 2010^10^, did not significantly impact the trend of AP numbers until quite recently. Indeed, in 2017, the number if APs started increasing again, to 26,869 positions (+4.3% over 2016), highlighting how a decrease of their use for teaching activities is not foreseen for the following years (see Figure 1).

Despite APs being the most conspicuous category of academic staff, they are disregarded by the official narratives and statistics of academic institutions as they are considered an exceptional circumstance which each department can manage individually. Representatives of academic institutions often justify this attitude by claiming that APs are “professionals with solid careers outside the academic system,” recalling the juridical definition of APs according to Law no. 382/1980, as if their juridical and social status have not changed in the last 20 years.

The results of both our survey and semi-structured interviews clearly disprove this narrative, showing how APs occupy unstable positions not only within the academic system but, in a number of cases, outside it as well, especially in the case of female APs.

In this section, we will focus on the outputs of the survey^11^, which was based on three main dimensions: (a) the academic career and (b) working conditions of APs, and (c), the ways they perceive their work. The survey was sent to almost 27,000 APs. From January 2018 to October 2018, we received 5,556 answers, covering more than 20% of the whole survey population. As a first step, we will examine the main structural variables of our sample and try to define a profile of APs, taking the gender variable as a point of comparison. As a second step, we will try to better understand the interplay between working conditions and experiences and professional aspirations, taking into account gender as a comparison parameter.

With respect to the whole AP population, the respondents to our sample are younger. Nevertheless, we can consider this is an effect of online surveys (see Table 1).

**Table 1:** Comparison of age of sample and age of total population (source: MIUR data and our survey).

	**Population (%)**	**Sample (%)**
<30 years old	2.40	2.10
30–39	23.50	31.10
40–49	28.40	31.30
50–59	23.60	21.00
>60 years old	20	13
N/D	2.30	1.10

Men were 54.4% of the respondents and 45.6% were women, with the majority working in the universities of northern Italy (59.1%), followed by those of central Italy (26.9%), southern Italy (12.8%), and distance learning/online universities (1.2%). Despite the official representation of APs as “successful professionals,” 48% of our respondents do not earn more than € 15,000 per year. Furthermore, 58% of these who earn < € 15,000 per year are women. Last but not least, among our respondents, more than half of the younger APs, female APs, and APs working in southern Italy earn < € 10,000.

**Figure 2:**
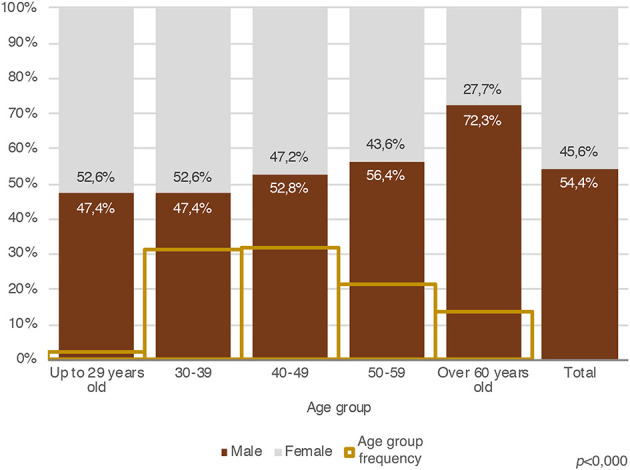
Gender distribution by age group and incidence of total age group (Source: our survey).

Focusing on female APs, Figure 2 shows that, up to the age of 39, women are the majority of our sample, whereas their numbers decrease progressively in the following age groups.

**Figure 3:**
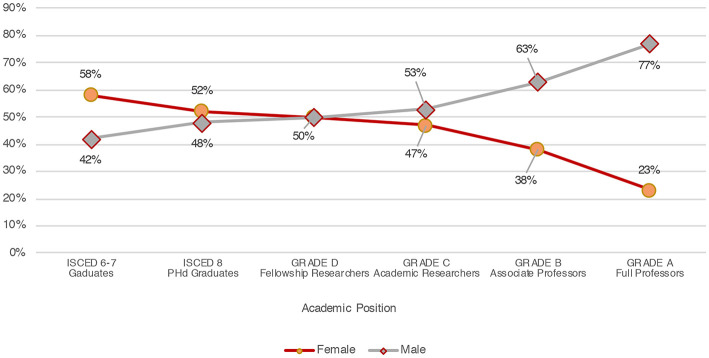
Proportion of men and women at each academic career step (Source: MIUR).

We can argue that the progressive decrease of women in older age groups is typical of the composition of Italian academic staff. Indeed, as the Ministerial data (data source: CINECA, March 2019) on women's academic careers demonstrate, in almost all disciplines (defined in Italy in terms of scientific disciplinary sectors, SSDs) the majority of students and PhD graduates are women. As stated above, they are also more than half of the research fellows (data source: CINECA, 2017). Conversely, their number decreases significantly and progressively when we look at the higher positions of the academic career: women make up 41% of senior researchers, 38% of associate professors, and only 24% of full professors (data source: CINECA, May 2019—see Figure 3).

To explain this trend, we can pinpoint some pivotal factors which generate gender discrimination in international academic contexts: first, enduring over time the uncertainty which characterizes academic careers may be heavier for women than for men, mostly considering gender discrimination regarding family responsibilities (Wolfinger et al., 2009)^12^; second, gender bias may persist within some SSDs (disciplinary scientific sectors) (Morgan et al., 2016); third, this gender bias may also depend on the gender discrimination women experience in the productive fields outside academia. In addition, it is important to consider that, in Italy, the high number of inactive women as well those volunteering part-time mainly depends on the fact they bear the primary responsibility for care activities in the domestic sphere. Thus, we can assume that women are more discouraged than men to pursue academic careers when their academic paths would be characterized by uncertainty, low wages, and short-term contracts.

**Figure 4:**
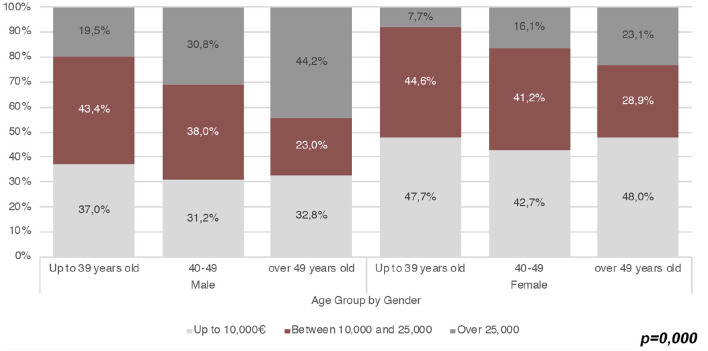
Wage range and distribution by age and gender (Source: our survey).

Figure 4 shows the distribution of wage ranges by sex and age. In all the age groups, the number of workers earning up to € 10,000^13^ is greater among women than men, whereas in all the age groups, the male rate of APs with a wage higher than € 25,000 per year is almost double the female rate. The difference by gender is even greater among younger APs earning more than € 25,000 per year, with an incidence of 19.5% for men and 7.7% for women. Furthermore, beyond having lower wages, female APs also have a more fragmented work experience. Indeed, if on average, the majority of APs who declare extra-academic work contracts are either self-employed (30.6%) or permanent employees, both categories are higher among men (34.1 and 23.2%) than among women (26.2 and 20.7%). By contrast, more women than men carry out informal work (20.1% vs. 17.4%), have fixed-term employment contracts (9% vs. 6.1%), or mixed forms of semi-employed contracts (24.1% vs. 18.8%).

**Figure 5:**
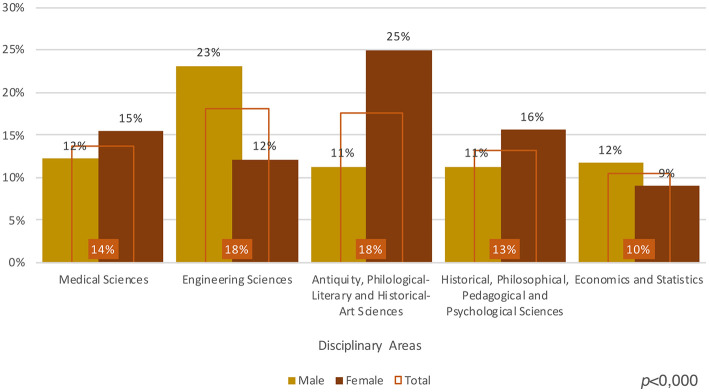
Scientific areas, incidence by gender and total (Source: our survey).

As mentioned above, for APs gender discrimination is reinforced by the gender segmentation which characterizes the SSDs. We have seen that women are a majority of students, graduates, and PhD graduates. Nevertheless, in the natural sciences, mathematics and statistics, information and communication technology, engineering, manufacturing and construction, women constitute 40% of graduates and 44% of PhD graduates. Furthermore, their number decreases in the subsequent steps of the academic career. This dynamic helps us to understand how gender segmentation in SSDs lead to the ejection of women from those areas which are more favored by the labor market^14^. This kind of segmentation also characterizes our sample. So, while 23% of male APs come from Engineering Sciences, 25% of female APs come from Classics, Philological-Literary Studies, and Art History. Similarly, the rate of APs in History, Philosophy, Pedagogy and Psychology is higher among women (16%) than men (11%) (see Figure 5).

Summing up, the comparison between the official data on academic staff and our sample of APs highlights similar patterns of gender discrimination: women are less involved than men in sectors which have a better recognition on the broader labor market.

Turning now to the interplay between work experience and the professional aspirations of APs, a key aspect to consider is the little interest given to them by academic institutions.

Official discourses which represent APs as outsiders with respect to the academic system are symbolically indicative of the precariousness of APs. While we can assume this perception is based on a lack of knowledge of the effective academic work of APs^15^, on the other hand, it also demonstrates a lack of recognition of their role. Our hypothesis is that the more APs participate in academic life, the more profound their feelings of being unappreciated may be.

**Figure 6:**
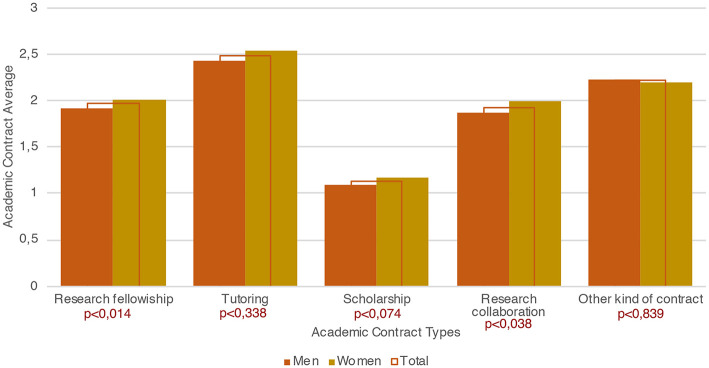
Work experience in the academic sector, average by gender, and total (Source: our survey).

If we look at the survey data, the results are quite interesting. First, 64.4% of the interviewees state that they had previous work experience at the university (see Figure 6 for details). Among female APs, however, the rate was 67.2%, whereas among male APs it was 62.2% (2-tiles correlation 0.01). Second, despite the women in our sample being younger than the men, they have a higher average number of university contracts than male APs. In addition, in the case of contracts as APs, women have a higher average number of contracts than men: 18.05 vs. 12.14.

However, if we use research activities as an indicator, we can observe a greater “dynamism” among male APs than female APs: so, in the previous 5 years, the men have published 11.51 works and participated in 9.43 conferences, on average, whereas the women have published 9.72 works and participated in 8.06 conferences^16^.

**Figure 7:**
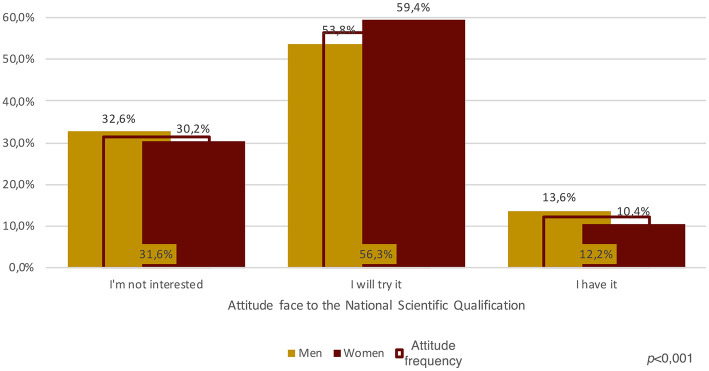
Attitudes toward the National Scientific Qualification, incidence by gender, and total (Source: our survey).

We believe that the greater attention male APs paid to producing research outputs may have an impact on their real chances of pursuing a linear academic career (Wolfinger et al., 2009), that is investing time for both increasing their scientific capital (publications) and their social capital (by participating in conferences) (cf. Bourdieu, 1984, 1986, 1989). This point is, in some ways, corroborated by attitudes toward the National Scientific Qualification (ASN): although more women than men state that they will try to attain the ASN (59.4% vs. 53.8%), more men than women have already received it (13.6% vs. 10.4% K^2^ 0,001 – see Figure 7).

In order to better identify the extent to which APs are interested in pursuing an academic career, we constructed an index of academic aspiration^17^. At a glance, the academic aspiration results are moderate for both men (38.4%) and women (37.6%). However, if we look at the two poles of the distribution, the percentage of male APs with the lowest academic aspiration score is higher than the percentage of women with the lowest score (28.4% vs. 25.3%) and, conversely, the percentage of female APs with the highest academic aspiration score is greater than the percentage of male APs with the highest score (37.2% vs. 33.2%)^18^.

With respect to our purpose of investigating how gender inequalities increase in correspondence with the worsening of working conditions, we can argue that the index of academic aspiration, is correlated to two crucial aspects of APs' working conditions: the fact part of their work is unpaid, and the fact they often work beyond the terms of their contracts.

By law (no. 313/2011), APs are only paid for their time spent lecturing in the classroom. Thus, according to the results of our survey, for each paid hour, on average, they also work: 0.56 h for office hours, 1.6 h for preparing lectures, 0.45 h for administrative tasks, 0.8 h for exams, 0.86 h for travel, 0.81 h for thesis and 0.2 h for further activities. As a result, for 1 h of lecturing, APs work 5.3 h for free. Thus, if on average for 1 h they are paid € 46.58^19^ (gross), they effectively earn € 6.71 (gross, and generally without additional costs for the university)^20^. To analyze the relationship between paid and unpaid hours, we constructed an index of “unpaid work.” When the relationship is lower than 2, we consider it a low intensity of unpaid work; when the relationship is between 2 and 3.5, we consider the intensity medium; finally, when the relationship is more than 3.5, the intensity is high.

**Figure 8:**
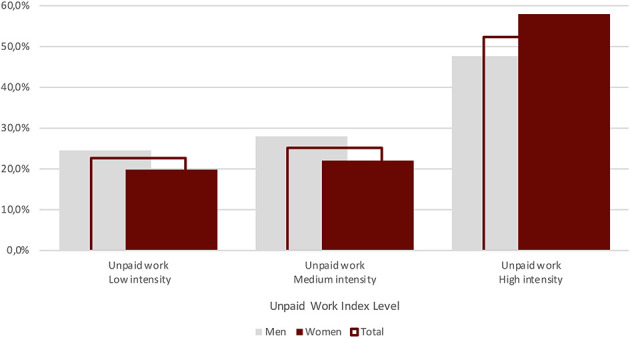
Unpaid work index, level incidence by gender and total (Source: our survey).

As Figure 8 show us, the majority of respondents (52.3%) present the highest intensity of unpaid work. However, female APs with a high intensity of unpaid work are 10% more than male APs. Furthermore, women work beyond the end of their contracts more than men (70.3% vs. 66.7%)^21^. Hence, the lack of economic recognition affects more women than men. This result raises meaningful questions about the fact that APs who are more engaged in teaching activities, while it seems they increase their participation in the academic life, on the other hand have less time to devote to research activities, therefore, to other forms of participation to the academic life, which are more symbolically acknowledged (cf. Heijstra et al., 2017).

**Figure 9:**
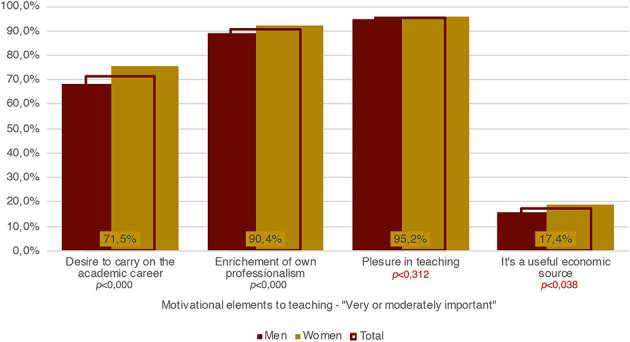
Motivational elements for teaching—“very or moderately important,” incidence by gender and total (Source: our survey).

As we have seen, the difference between women and men in terms of unfair working conditions may be explained by a different degree of motivation to pursue an academic career. Nevertheless, to the questions concerning their motivations for working as APs, they answer similarly. Generally, both men and women give great importance to the pleasure of teaching (nearly 100%), and both men and women consider the AP experience important for enhancing their professional competences (nearly 90%). We can observe some slight differences only with respect to the desire to pursue an academic career, which is higher for women than men (probably depending on the higher average age of men), and economic motivation, which is also higher for women than men (Figure 9). The latter result is probably the most interesting, since women are, in general, more vulnerable than men to the lack of economic recognition in the labor market. Thus, we can assume that, on average, every additional source of income is more important for women than for men. In the next section, by analyzing the semi-structured interviews, will also try to better investigate whether, and in which ways, the different economic importance given to their work as APs influences their acceptance of their working conditions.

**Figure 10:**
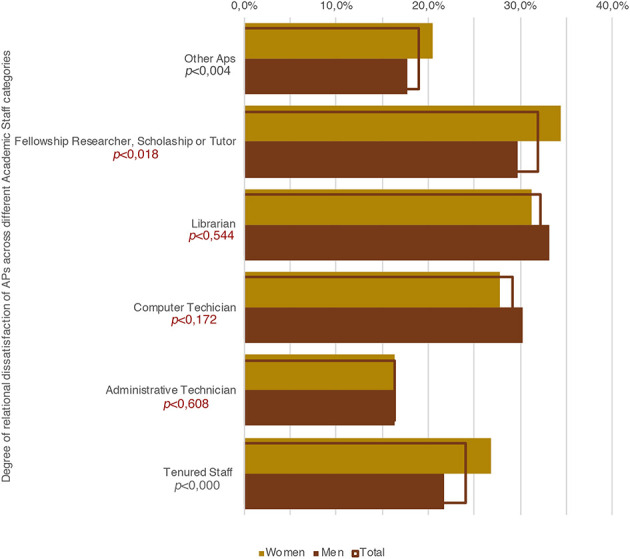
Degree of relational dissatisfaction of APs across different academic staff categories.

A final aspect we considered, and which we will more fully explore in the next section, concerns the academic relationships of APs with the academic staff. As Figure 10 shows us, women have generally more difficulty interacting and constructing relationships with professors and researchers with both tenured and fixed-term positions. Conversely, their relationships with the administrative staff (mostly female, 58.8%, data source: USTAT-MIUR) are better than men's relationships with the administrative staff. Also considering the previous results, two aspects may be considered as meaningful. The first concerns the fact that female APs are more engaged in teaching duties than male APs. However, despite this engagement, their social relations within academia are poor; or, in other words, they endure a greater condition of social isolation and “invisibility” (Honneth, 2003). This aspect also reinforces the idea that teaching activities are less important for building “social capital” (Bourdieu, 1986) than research activities. The second aspect concerns the fact that female APs find it easier to construct relationships with the administrative staff than with professors and researchers, that is, with people who enjoy less prestige, meaning those who have less scientific and academic capital. Thus, from a Bourdieusian perspective (Bourdieu, 1984, 1986, 1993), beyond the fact that they juridically occupy the same position as male APs, on average, the objective position of female APs employed within the located social space of the academic field (i.e., the institutes where they work) is lower than the position occupied by male APs, at least in terms of the amount and composition of their capitals.

## Experiencing Academic Life: A Gender Perspective

In this last section^22^, by adopting a gender perspective we will look more in detail at how female and male APs interpret their academic experiences, position within the academic field, and academic/professional identities. To achieve this objective, we carried out 31 interviews with APs from northern (20), central (9), and southern (6) Italy^23^, and from different disciplinary macro-areas (Humanities: 11; Political and Social Sciences: 9; Natural Sciences: 3; Medicine: 3; Law: 2; Engineering: 3)^24^. Fifteen of our interview-partners were women and 16 were men. In terms of age, seven of them were over 55 years old, seven between 45 and 55 years old, and 16 under 45 years old^25^ (see Table 2). Further data we considered to be crucial for understanding the ways in which APs perceive their academic experiences are: whether they carried out extra-academic activities (26) or had additional fixed-term contracts with universities (5), and the number of years they had been working as APs. This last information is especially useful to understand whether they had experienced the academic system before the last university reform (2010), which radically changed the career path and structure in academia.

**Table 2:** Distribution of interview-partners by gender and age.

**Gender/Age**	**<45**	**45–55**	**>55**
Men	8	3	5
Women	9	4	2

Nevertheless, although this information can be viewed as pivotal for determining the ways APs experience academic life and narrate this experience, the comparative analysis is not strictly dependent on one or another of these factors. Rather, by comparing the narrative structure of each interview, we try to identify the underlying structure of meanings (cf. Demazière and Dubar, 1997) in order to understand how APs perceive their academic careers and which kinds of *academic identities* (cf. Henkel, 2005; Leisyte, 2015) they have developed over time.

In this regard, the gender perspective we adopted did not condition the interpretation itself. Indeed, if the survey highlights meaningful differences between men and women's working conditions, as well as differences in judging their degree of integration within academic life, we do not assume *a priori* that these differences deterministically influenced the ways individual APs narrated their own professional paths. The main question we have asked was, instead, whether it is possible to pinpoint a different academic/professional habitus between male and female APs. While this habitus depends on the structural position APs occupy in the wider academic field, it also influences the different ways they perceive and think about their positionality within the academic field.

To answer this question, we have considered three significant aspects. The first concerns the time structure of the narrated experience (cf. Berger and Berger, 1972); the second concerns the different levels of recognition or non-recognition felt by the interview-partners; the third concerns the global view they have of the academic system. With regard to the first aspect, we have examined how APs understand and reconstruct both their work routines and the relationship between their academic experiences and aspirations. With regard to the second aspect, we have looked at the interplay between self-representation and the representation by others in the formation of their own *professional social identity* (cf. Dubar, 2000). Thus, we have focused on how APs define, classify and evaluate their own scientific/academic competences and express their feeling of belonging within academia. Finally, the third aspect concerns the different interpretation of formal and informal academic dynamics. Indeed, the different ways of understanding these dynamics conditions the practices and strategies APs have adopted for constructing their own professional social trajectories.

Summing up, we assumed that a better understanding of each interview was possible only by taking into account these three aspects together. This analytical procedure allows us to dig into the deeper layers of meaning in each narrative, in spite of the fact they put forward similar topics in similar ways. So, for example, all the interview-partners claimed that the remuneration for their work is disproportionate to the time they employ for the work itself, data, as we have seen in the previous section, that is important to grasp their concrete working conditions. Furthermore, all the interview-partners maintained that, despite this disproportionality, they make every effort to prepare their lessons optimally. Indeed, all the interview-partners (according to the responses of the survey – see Figure 9) assert that the main satisfaction of their work is derived from relationships with their students and from the pleasure of teaching itself. Not least, all of them declared that they go to their departments almost exclusively when they have class, exams, or office hours, mainly for two reasons: either because they have no place to stay or because they carry out many working activities in order to live and it is difficult to organize their working time so as to spend more time at the institute outside of their required activities.

Hence, if we examine the individual answers APs gave on specific issues, we can claim that male and female APs experience their work in very similar ways. However, if we consider instead the whole narrative of their “academic/professional life,” several differences emerge with respect to these points.

The main contrasting aspect among male and female APs concerns the fact that the former declare that, though they are more involved in the research activities of faculty members, they are also more frustrated at being excluded from the political decision-making procedures of their institutes. Furthermore, some of them declare that they have reduced their presence at university over time because of a sense of unease they feel after working as APs for years without any prospects of stabilization. This answer has been mainly given by older male APs^26^. However, if we compare the interviews of the corresponding age group of female APs, we do not notice a similar feeling. More interesting is the fact that six of the male APs belonging to the older age group are either independent professionals or permanent employees outside of academia (see footnote 15). Hence, while their social and economic status are apparently ensured by their extra-academic profession (engineer, lawyer, doctor, etc.), they consider their academic work as primary for their professional identity. Indeed, in their professional identity narratives, they stress how, since taking their degree, they have had a continuous relationship with the university (in some cases for more than two decades).

In the case of the older female APs, we do not observe such a long-term relationship with the university, even though most of them point out that they began working as APs after finishing their PhD dissertations. Furthermore, on average, their extra-academic activities are less prestigious than the extra-academic activities of the older male APs. Nevertheless, their interviews highlight how the uncertain prospects for the short and long-term oblige them not simply to find additional work to economically support their desire of pursuing an academic career, but to search for jobs that could become an alternative professional strategy. This double professional strategy, in addition to the awareness of occupying a marginal position, leads them to feel that they only partially belong to the academic world.

Thus, from this perspective, if we compare the whole sample of interviews, we find more affinities if we consider the gender variable rather than age. So, for instance, even the younger female APs express a similar “strategic perspective” of constructing more than one professional path. Furthermore, they make clear how most of them have been following this double professional strategy since receiving their undergraduate or PhD degree. It is probably not the case that more younger female APs than younger male APs have obtained permanent employment outside academia (in either the public or private sector). What is not taken-for-granted, however, is that this job also constitutes, in some cases, their main professional identity. On the other hand, this position is also shared by those younger female APs who are or have been research fellows. By explaining, then, to what extent they feel they belong within academia, they restrict their membership to the specific activities they carry out and to the temporal limit of their contracts. Some of the older female APs even emphasize that, while they feel they do belong to the university where they work, they do not feel part of the scientific community, implying that belonging to it would require further qualifications and crossing institutional boundaries.

Turning now to the interviews of the older and younger male APs, we also notice an ambivalent feeling between being included in and excluded from academia. What differs from the women's narratives are the different temporal meanings given to this feeling, the foundation of their self-image as academics, and the image attributed by others from within the “institution” (colleagues, administrative staff, students) as members (or not) of “academia.” Thus, more than their female counterparts, male APs claim they possess *personal* scientific competences which are often underestimated by the “institution” or the “system” (the economic parameter is also crucial, see footnote 22), even though they are recognized by their colleagues (permanent and non-permanent staff). Part of these competences, however, cannot be objectified. Thus, differently from women, who pay more attention to reaching the objective criteria defined by the universities for “measuring” their competences both in teaching and research, male APs believe that their value as “scholars” or “professors” cannot be reduced to these objective criteria. Moreover, some of them consider these criteria as penalizing their experience within the university, which is defined not only by their past and present teaching activities, but also by their higher tendency and intention to participate in the whole academic life. Hence, having constructed their academic identity on the basis of the time they have spent over the years working at the university, they perceive their “exclusion” from it more deeply than women, who mainly base their sense of belonging on their “temporary” routines^27^. So, the sense of “exclusion” acquires different meanings between male and female APs. For male APs, it is strongly related to a still missing, and probably never arriving, stabilization of their “position,” which could ensure their academic identity. For female APs, instead, it is more related to their everyday academic practices and relationships, which are often negative or inexistent^28^.

Almost all the female APs mention negative episodes they experienced in the workplace, which in turn highlight three kinds of negative relationships. The first concerns a general disregard on the part of the permanent staff toward their difficulties in carrying out their jobs, that is, of completing the same tasks as the tenured professors, despite their very different working conditions. The second concerns a higher perception of the existence of academic power relationships. If these power relationships structurally depend on the different positions that tenured and contract professors occupy, they are also practiced and performed daily, limiting in part the teaching autonomy of APs. The third concerns “competition” either among peers (APs and/or research fellows) or with those who have recently obtained a tenured position. Despite these negative experiences, in their narratives they stress how they cognitively and practically react by creating emotional distance from the academic world. As a result, they also have a less idealized concept of academic life and relationships than male APs (see, for instance, footnote 28). Those who are still active in research state that they are part of scientific networks which they construct mainly outside the university where they teach. Not less, they also add that they see these networks as temporary until they have the economic possibility of carrying out research activities (publications, participation in conferences and seminars, etc.). Finally, differently from male APs, female APs more strongly emphasize a lack of collaboration and organization in their degree courses. In this way, they also stress again how their position and life as APs are strictly related to the concrete activities they carry out and to the concrete relationships they establish for carrying them out. In the case of male APs, in contrast, it seems that the “exclusion” they feel is mainly a personal question, that is, it is more related to the fact that they still have not obtained a tenured position.

Thus, to move toward our conclusions, whereas factually both the survey and the semi-structured interviews, supported by the international literature we reviewed, confirm that women's experiences of academic life are more negative than men's, the latter express more negative feelings toward their conditions. This stems from the fact that they have invested more personally in academic life as the sphere of their self-realization, even though they possess more prestigious positions outside academia than female APs. Furthermore, the aspirations that male APs have to pursue an academic career are more supported by the presence of informal relationships, at least ideally. Conversely, women measure their possibilities of pursuing an academic career on the basis of both objective evaluation criteria and the *objective power relationships* which structure the specific academic or disciplinary fields in which they participate (cf. Bourdieu, 1984), that is, they are also more adherent to an objective understanding of career (cf. Hughes, 1958). Thus, whereas men see their lack of stabilization as a sign that undermines their whole identity as a person, women consider it more as the result of the logic of their academic/disciplinary fields, that is their general more precarious economic condition, which also influence on their temporal strategies, result in a sharp knowledge of the “career realities” (cf. Hughes, 1958, p. 128). As a result, for men the lack of recognition they experience as APs is more difficult for them to accept than for women.

On the other hand, male APs have a stronger “sense of belonging” than female APs, precisely because this feeling is not based on their temporary working conditions, but on their interior feeling of “being made” for teaching and researching. This belief makes it probable that male APs are more inclined to expect something and to continue to work at the university, until at least, as someone of them stressed, they are able to sustain these working conditions more psychologically than economically. Conversely, female APs, when they decide to stop (or are considering stopping) working at universities, put as the first reason a lack of time and the fact that this job causes a lot of physical stress, which also depends on the fact that they carry out additional work activities (often in different cities). Furthermore, more than male APs, they show that they have been more aware since they started as APs that this activity would not be very helpful for their academic careers, apart from the possibility of building their CVs.

In short, whereas male APs perceive a discrepancy between their self-image and the position they occupy, female APs adopt a viewpoint more in line with that of the institutions in order to understand, practically, what realistic possibilities they have within academia and which professional strategies they can pursue.

## Conclusions and Discussion

In this article, we have tried to highlight specific forms of unpaid work, by looking at non-standard works within academia and by taking Italian APs as a paradigmatic case study. This choice was also due to the increasing split within the national and international academic market between research and teaching positions in terms of economic and social status, as well as job security. In this regard, we carried out a survey which reached 5,556 Italian APs, and we conducted semi-structured interviews in order to better understand their working conditions and how these working conditions influence both their concrete academic careers and aspirations.

We decided to conduct an analysis from the perspective of gender in order to investigate whether and to what extent differences among male and female APs can be observed even at this lowest social order of the academic structure. On the other hand, we consider the research results also to be meaningful for a wider reflection on the problematic role that women still occupy in the academic market, as well as in other productive fields (in Italy as well as abroad), especially those related to intellectual professions and informal forms of work.

The survey highlights how female APs are more exposed to the risk of unpaid work, earn less on average, and have a more fragmented work experience (considering both their academic and extra-academic activities) than male APs. Thus, from an economic and temporal viewpoint, they are more disadvantaged than men for investing in building an academic career. On the other hand, a further striking output of the survey is that female APs invest more time in teaching duties than their male counterparts, whereas their research outputs are inferior to the those of the latter. While this might depend on the fact that, in our sample, the male APs are older than the female APs, on the other hand, the more time the latter spend on teaching and the more their economic conditions oblige them to work at other jobs, the less time they have to devote to research. Two further indicators we considered in analyzing the data were the “degree of relational satisfaction within academia,” and the degree of academic aspiration. With respect to the former, it is clearly evident that women perceive the academic milieu as unfriendly more than men do. With respect, instead, to the latter index, we did not find meaningful differences.

The semi-structured interviews have been useful for an in-depth analysis of the meanings that male and female APs give to their jobs in order to better understand how their different working conditions concretely influence their academic aspirations. The main remarkable result is that female APs seem less interested than men in pursuing an academic career. In their narratives, women stress a more practical view of their position within the academic system and their academic work, which in turn derives from their economic conditions and more acute perception than men of the social structure of power relationships in their workplaces, which in Italy are strongly influenced by a familistic culture. As a result, it seems that women more than men, look for job opportunities outside of academia. Conversely, men seem to have a more idealistic vision of their working and being at the university, that is they have deeper interiorized the academic *illusio*, which is more based on a traditional (and familistic) view of the academic life, formally modified by the University reforms of the last four decades. Thus, independently from their economic and social status outside academia, male adjunct professors interiorize a greater sense of vocation to the academic profession than women, which is therefore pivotal in forming their habitus and orienting their practices. This sense of vocation seems, at the same time, to be supported by the fact that they feel more integrated than women in the workplace, which may also depend on a greater temporal (and mental) investment in academic relation and life. On the other hand, however, their stronger academic identity causes them more frustration when facing a perceived lack of recognition which they interpret as the lack of the possibility of obtaining a permanent position.

## Data Availability Statement

Due to the outlined ethical reasons, the dataset generated by this study is not publicly available. However, other datasets used in this study can be found in USTAT-MIUR (http://dati.ustat.miur.it/organization/ace58834-5a0b-40f6-9b0e-ed6c34ea8de0?tags=Universit%C3%A0&tags=Personale) and CINECA (https://cercauniversita.cineca.it).

## Ethics Statement

In Italy, no local legislation was in effect when the authors began the survey. Furthermore, the authors did not consider it necessary to require an ethical review process since, for social research of this kind, neither the University of Bologna nor Bicocca required ethics committee approval, and no personal or sensitive data have been asked of the survey respondents. Individuals gave their consensus to participate in the survey by responding to a specific question, after having been informed about researcher contacts and purpose, and were assured that data would be anonymized and used only in aggregate and anonymized forms. The program we used for the survey allows the anonymization of the IP.

## Author Contributions

This article is the result of a collaborative common research work. We declare that BG wrote the introduction, experiencing academic life: a gender perspective, and conclusions and discussion sections. GD wrote the subjectivities at stake: the other side of non-standard work, academic careers in the neoliberal university, and the careers of APs: a general overview sections.

### Conflict of Interest

The authors declare that the research was conducted in the absence of any commercial or financial relationships that could be construed as a potential conflict of interest.

## Footnotes

^1^In Italy, adjunct professor (*professori a contratto*) is a type of academic appointment in higher education. It is a non-tenure-track position and, by law, APs may have only a 1 year contract, which cannot be renewed. Nevertheless, there is no temporal limit for the number of years an AP can teach at the university. As a result, a conspicuous number of APs who answered to our survey have taught for several years at university, but every year they participate in an academic competition for a position as AP. From a juridical point of view, APs are not employed by universities but are hired as external collaborators.

^2^In this regard, many biographies of well-known artists, academics, and writers which are generally structured on the opposition between the geniality of the individual and their adverse working conditions and destiny until their consecration after death.

^3^All the interview-partners used pseudonyms.

^4^There is no corresponding English word for the French *dispositif* used by Foucault and Gordon (1980), the Editor uses the Latin word *apparatus* beside the French word (Foucault and Gordon, 1980).

^5^The *assistente volontario* (volunteer assistants) were not paid and generally helped “their” professors in teaching activities (support during exams, tutorials, etc.). Law no. 1962/1967 abolished this figure and introduced the fixed-term positions of “research fellows” and post-graduate “research assistants” (*borsisti* and *assegnisti*).

^6^Until the 1990s, there was a specific configuration of university networks. Thus, the bigger universities played a central role in informal recruitment policies, whereas smaller and peripheral universities were under the control of the biggest ones. As a result, in the smaller universities, the permanent staff was continuously changed, with consequences for the organization of teaching and research activities (cf. Moscati, 2001).

^7^Art. 25 of Law no. 382/1980.

^8^As we will show in the following sections, the last juridical changes have brought meaningful social consequences not only with respect to the working conditions of precarious scholars but also the temporal structure of the academic path, in terms of both the possibility of imaging an academic path and the ways of experiencing daily the academic life. This point is especially noteworthy, if we compare the position of the “liberi docent” (similar to the position of the German *Privatdozenten*, cf. Schultheis, 2000) with that of the adjunct professors. If also the “liberi docent” experienced social and economic precarious conditions within the Academia, they were structurally more integrated in the academic milieu. Indeed, taking the “libera docenza” was noteworthy for achieving an academic career. Conversely, cumulating experiences as adjunct professors nowadays has little relevance. This means, not least, that for the “liberi docent” waiting with the hope of achieving a tenured position was stronger related to concrete social expectations than for adjunct professors.

^9^Due to the juridical framework of the Italian academic structure in which, for example, it is needed to accede to competitions for positions of tenure-track junior professor (a figure introduced in 2010, replacing the previous figure of the tenured lecturer), we consider here the position of research fellow as “privileged” for pursuing an academic career.

^10^Both junior and senior researcher positions last three years. For junior researchers, teaching duties are optional. Senior researchers have to teach and, after the national scientific qualification, they can automatically advance to the position of associate professor.

^11^The research was conducted in compliance with the national regulations governing the protection of personal data (Legislative Decree 196/2003 and following regulations), with the request for an online respondent's informed consent and the authorization to fill in the questionnaire and to process the results ensuring privacy preservation by data anonymization.

^12^This aspect is especially evident in the interviews with female APs. Paradigmatic is the interview with a 55 years old AP who has taught for 12 years in four different universities. To the question about why and how she decided to work as an AP, she answered: “I started working as an adjunct professor after participating in an academic competition for a lecturer position, which had not ‘been intended’ for me. Working as an adjunct professor has been a sort of compensation. During this period, I have also participated in competitions for research fellow positions, but once I was asked to withdraw my application and it was made clear to me that I was needed as part of the work force but could not aspire to an academic career.” To the following question about “extra-academic activities,” she answered: “I teach at school, otherwise I could not survive.” As we highlight in this section, most female APs carry out traditional female work activities which are usually less prestigious and lower paid than working activities carried out by men. Furthermore, more women than men reported episodes of mobbing in their academic career, highlighting in this way how, in Italy, the phenomenon of “amoral familism” is especially unfavorable for women [translation by Barbara Grüning].

^13^We considered here all the jobs carried out by the respondents, both within and outside academia.

^14^Following the Almalaurea data on the work experience of PhD graduates in 2018, we can observe that, one year after receiving a PhD in Engineering, 37.1% of workers had a non-fixed term contract, whereas after a PhD in the Humanities the non-fixed term contract rate was 25%. Moreover, among PhDs in the Humanities, 29.6% had non-standard contracts, while only 17.6% of PhDs in Engineering had non-standard contracts (https://www.almalaurea.it/sites/almalaurea.it/files/docs/universita/postlaurea/dottori_occupazione_report2018.pdf).

^15^In particular, in various conversations with representatives of the governance of the University of Bologna, APs have been depicted as professionals who are not oriented toward an academic career. Thus, beyond the legal definition of APs that excludes them from the official faculty, it emerges how in the practices and discourses originating from the university's governance, APs are not considered either for the present or for the future as members of the academic staff. This aspect is also stressed by the various university reform proposals which have been carried out in the recent years by both politicians and academics, which in fact have not brought any improvements for APs as they mainly focus only on academic research activities. Thus, these social discourses highlight the practical effect of the legal separation of teaching and research activities at the lower stages of the academic structure.

^16^We calculate the statistical average on the basis of a distribution of outputs from 1 to 201. The standard deviation of the distribution corresponds to 16.8 and 13.7, respectively, for the publications and conferences of the male APs and to 12.01 and 9.7 for the publications and conferences of the female APs.

^17^The index was calculated as the sum of answers with respect to the desire to pursue an academic career (Agree Strongly = 3, Agree Moderately = 2, Agree Slightly = 1, Disagree = 0) plus answers about their attitude toward the ASN (Not interested = 0; Interested = 1; Have qualification = 3) minus the existence of previous contracts with academic institutions (Yes = 1; No = 2). In order to reduce the scale of the index, we synthetized the results into three types: Low academic aspiration (score between 1 and 2), Moderate (between 3 and 4), Strong (between 5 and 6).

^18^The index distribution is not significantly correlated to the gender (K^2^ = 0,025). Nevertheless, our purpose is not to identify those APs with high academic aspiration. Rather, it's an useful instrument for investigating what happens to those APs with the highest academic aspiration.

^19^Excluding errors and missing data, the average was calculated from data provided by 3,131 APs.

^20^Depending on the kind of contract, net earnings are 22 or 33% lower than the gross.

^21^The relation is significant at 0,000 (K^2^).

^22^The qualitative analysis is here mainly considered as support for better interpreting the findings of the quantitative analysis. We refer to a further article in preparation, where more space is devoted to the interviews.

^23^Since some of the APs worked or are working in more than one university, we have considered them twice here. For what concerns more generally the geographic cultural dimension of the Italian Universities, we have not analyzed in depth how this produced meaningful consequences on the career paths. Nevertheless, two observations are noteworthy. First, in the last decade the universities of Southern Italy have more suffered from the relevant cutbacks to the financing of public universities. This had a twofold consequence: on the one hand there are her fewer permanent positions than in northern Italy, on the other hand, however, there are also fewer tenured positions, since Universities in Southern Italy have difficult also to fund them. Thus, scholars who aim at working in Academia should often working (completely) for free as “volunteer assistant” or “cultori della materia.” This entails, not least, that in these situations the familistic academic culture is stronger than in Northern Universities, as also some of the interviews highlight. The second point concerns the peripheral and isolated position of many universities of Southern Italy. As a result, precarious academic workers find here greater difficulty to build academic networks, which could increase their opportunities in the academic market and the possibility of pursuing an academic path outside the original academic milieu (cf. Giancola et al., 2016).

^24^It is important to note here that, proportionally to the disciplinary macro-areas, most of the APs are either humanists or social/political scientists.

^25^We have chosen this kind of age division since, in Italy, on average people reach tenured positions when they are older than 40 years old.

^26^So, for example, the striking answer of a 59 year-old male AP to the question about the main positive and negative aspects of working as an AP: “The gratification does not derive from the legal or economic recognition of the work. The damage consists in the frustration of fulfilling high office, without having any recognition for your career.” To the question about his feeling part of academia, he stressed that “it is intermittently psychologically devastating.” This aspect also emerges in the ambivalent relationship with his colleagues: “I'm respected by my colleagues, but in general, as an adjunct professor, you are considered ‘a child of a lesser God”’ [translation by Barbara Grüning].

^27^So, for example, the answer of a 49 year-old female AP about her relationship with her colleagues: “usually I meet my colleagues who have a tenured position at conferences. I can say that our relationships are very good. Well, they all know my desperate situation and that I will probably never get a tenured position. Nevertheless, I don't have to thank them for anything, and they don't owe me anything. So, the relationship is friendly” [translation by Barbara Grüning].

^28^In this regard, the answer of a 42 year-old female AP to a question about positive and/or negative episodes concerning her work within the university, is paradigmatic: “I'd like to start with a negative episode. I will never forget when a professor—nowadays a full professor, with whom I was working at the time in a University in southern Italy—told me “Grazia” [author's note the name of the AP has been changed] you may be both smart and qualified, but academically speaking, you are an orphan, so you cannot go anywhere” [translation by Barbara Grüning].
