# Corrigendum: Gender Inequality in Precarious Academic Work: Female Adjunct Professors in Italy

**DOI:** 10.3389/fsoc.2020.00031

**Published:** 2020-05-19

**Authors:** Gianluca De Angelis, Barbara Grüning

**Affiliations:** ^1^Department of Sociologia e Diritto dell'Economia, University of Bologna, Bologna, Italy; ^2^Department of Sociologia e Ricerca Sociale, University of Milan Bicocca, Milan, Italy

**Keywords:** unpaid work, adjunct professor, academic career, gender inequalities, Italy

In the original article, there was a mistake in the legend for Figure 4. The second wage range is Between 10,000 € and 25,000 €, so the third is Over 25,000 €. The same error affects the text describing the figure. The correct legend appears below and a correction has been made to the **The Careers of APs: A General Overview** section, paragraph 10:

**Figure 4:**
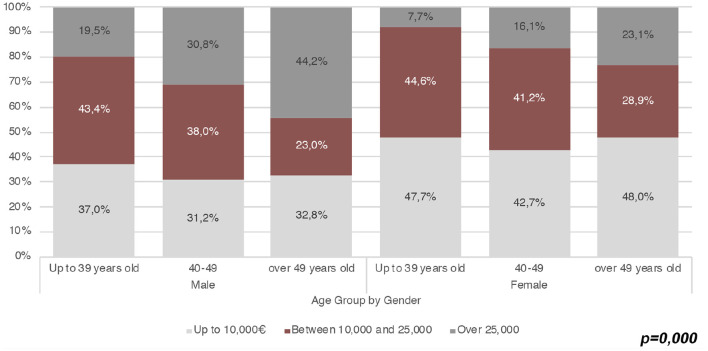
Wage range and distribution by age and gender (Source: our survey).

“Figure 4 shows the distribution of wage ranges by sex and age. In all the age groups, the number of workers earning up to € 10,000^13^ is greater among women than men, whereas in all the age groups, the male rate of APs with a wage higher than € 25,000 per year is almost double the female rate. The difference by gender is even greater among younger APs earning more than € 25,000 per year, with an incidence of 19.5% for men and 7.7% for women. Furthermore, beyond having lower wages, female APs also have a more fragmented work experience. Indeed, if on average, the majority of APs who declare extra-academic work contracts are either self-employed (30.6%) or permanent employees, both categories are higher among men (34.1 and 23.2%) than among women (26.2 and 20.7%). By contrast, more women than men carry out informal work (20.1% vs. 17.4%), have fixed-term employment contracts (9% vs. 6.1%), or mixed forms of semi-employed contracts (24.1% vs. 18.8%).”

The authors apologize for this error and state that this does not change the scientific conclusions of the article in any way. The original article has been updated.

## Footnotes

^13^We considered here all the jobs carried out by the respondents, both within and outside academia.

